# Wet-Spun Composite Filaments from Lignocellulose Nanofibrils/Alginate and Their Physico-Mechanical Properties

**DOI:** 10.3390/polym13172974

**Published:** 2021-09-01

**Authors:** Ji-Soo Park, Song-Yi Han, Rajkumar Bandi, Eun-Ah Lee, Azelia-Wulan Cindradewi, Jeong-Ki Kim, Gu-Joong Kwon, Young-Ho Seo, Won-Jae Youe, Jaegyoung Gwon, Chan-Woo Park, Seung-Hwan Lee

**Affiliations:** 1 Department of Forest Biomaterials Engineering, College of Forest and Environmental Sciences, Kangwon National University, Chuncheon 24341, Korea; pojs04@kangwon.ac.kr (J.-S.P.); laa3158@kangwon.ac.kr (E.-A.L.); azeliacindradewi@gmail.com (A.-W.C.); panda20@kangwon.ac.kr (J.-K.K.); 2 National Institute of Forest Science, Seoul 02455, Korea; sngkgk@korea.kr (W.-J.Y.); gwonjg@korea.kr (J.G.); 3 Institute of Forest Science, Kangwon National University, Chuncheon 24341, Korea; songyi618@kangwon.ac.kr (S.-Y.H.); rajkumar.pgcb@gmail.com (R.B.); gjkwon@kangwon.ac.kr (G.-J.K.); 4 Kangwon Institute of Inclusion Technology, Kangwon National University, Chuncheon 24341, Korea; 5 Department of Advanced Mechanical Engineering, Kangwon National University, Chuncheon 24341, Korea; mems@kangwon.ac.kr

**Keywords:** lignocellulose nanofibril, alginate, wet-spinning, composite filament

## Abstract

Lignocellulose nanofibrils (LCNFs) with different lignin contents were prepared using choline chloride (ChCl)/lactic acid (LA), deep eutectic solvent (DES) pretreatment, and subsequent mechanical defibrillation. The LCNFs had a diameter of 15.3–18.2 nm, which was similar to the diameter of commercial pure cellulose nanofibrils (PCNFs). The LCNFs and PCNFs were wet-spun in CaCl_2_ solution for filament fabrication. The addition of sodium alginate (AL) significantly improved the wet-spinnability of the LCNFs. As the AL content increased, the average diameter of the composite filaments increased, and the orientation index decreased. The increase in AL content improved the wet-spinnability of CNFs but deteriorated the tensile properties. The increase in the spinning rate resulted in an increase in the orientation index, which improved the tensile strength and elastic modulus.

## 1. Introduction

The modern world is highly dependent on petroleum resources [1]. Various industrial products are being manufactured from petroleum [2], and these petroleum-derived plastics are not biodegradable, resulting in environmental pollution. In particular, recently, the pollution associated with microplastics from petroleum-derived plastics and fibers has become very serious [3]. Microplastics are small plastic particles with sizes less than 5 mm, which threaten not only marine life but also human health through the food chain [4,5]. 

Biodegradable or biomass-based materials have gained increasing attention [6]. Lignocellulose is a biomaterial that can be obtained from wood, plants, and agricultural products, and it is an abundant, biodegradable, and sustainable material [1,2,3,4,5,6,7]. Its utilization as an environmentally friendly biodegradable material and as an alternative to petroleum-based materials has also attracted attention. In particular, nanocellulose obtained from lignocellulose is considered a highly promising material.

Nanocellulose is a cellulosic nanomaterial with nanoscale dimensions [8]. Cellulose nanofibril (CNF) is one type of nanocellulose, mostly obtained by defibrillation using mechanical defibrillation equipment such as a high-pressure homogenizer (HPH), wet-disk milling, ball milling, and ultrasonication. They have a diameter of 15–30 nm and a length in the micron-scale [9,10,11,12,13]. Lignocellulose nanofibril (LCNF) with lignin and hemicellulose contains hydrophobic lignin. Thus, it normally shows lower viscosity in water suspension and better compatibility with hydrophobic polymers than CNFs without lignin [14,15]. These characteristics can contribute to improving the processability and applicability of LCNFs.

Wet-spinning is a promising method for filament production from CNF suspensions [16,17,18,19,20]. The wet-spun CNF filaments can provide some advantages such as low cost, biodegradability, compatibility, high strength, and high applicability to advanced materials [19,20]. In the wet-spinning process using CNF suspensions, a solvent with low polarity, such as acetone, can be used as the collection solvent to obtain a filament with better shape. In a solvent with low polarity, the dispersibility of spun CNF can be suppressed, resulting in the improvement of hydrogen bonding among individual CNFs. This can cause the coagulation of CNFs to form filaments in the collecting solvent with low polarity. However, there is concern that LCNFs with hydrophobic lignin can deteriorate the wet-spinnability and coagulation in collecting solvents because of lower hydrogen bonding strength among LCNFs. Park et al. [17] reported that the hydrogen bonding in LCNF is weaker than in holocellulose nanofibril (HCNF) and PCNF, resulting in the deterioration of the coagulation of LCNFs for the formation of filaments. Thus, in order to improve wet-spinnability, it is necessary to use a binder that contributes to the coagulation of LCNFs.

Alginic acid is a linear copolymer of seaweeds. It consists of homopolymeric blocks of (1→4)-linked β-D-mannuronate (M) and *α-L*-guluronate (G): for example, consecutive G-residues (G-blocks), consecutive M-residues (M-blocks), and alternating M and G residues (MG-blocks) [21,22]. Sodium alginate (AL) is a sodium salt of alginic acid. It is widely used in many industries including food, textiles, pharmaceuticals, and biomedical materials [23,24,25,26]. In particular, it can be easily formed into beads or filaments by gelation. In the presence of divalent cations such as Ca^2+^, the sodium salts are replaced with Ca^2+^ ions, and the Ca^2+^ ions mediate ionic binding between guluronic acid blocks (G-blocks). As a result of this ionic binding, a three-dimensional network structure is formed. Thus, gelation of AL can occur [27,28,29]. The AL gel has attractive properties such as low cost, biocompatibility, biodegradability, and non-toxicity [27,30,31]. It has high viscosity owing to its three-dimensional bonding structure and is expected to have good affinity with LCNFs. Therefore, the gelation of AL in LCNFs can improve the coagulation of LCNFs into filament formation, resulting in the improvement of the wet-spinnability of LCNFs.

In this study, LCNFs with different lignin contents and PCNFs as a control sample were used for filament production via wet-spinning. The wet-spinning suspension was prepared by mixing the CNFs and AL that were wet-spun into CaCl_2_ solutions for gelation of AL. The effect of AL addition and wet-spinning conditions on the properties of composite filaments was investigated.

## 2. Materials and Methods

### 2.1. Materials

Korean red pine (Pinus densiflora S. et Z.) was obtained from the Experimental Forest of Kangwon National University (Chuncheon, Korea). The degreased wood powder was prepared using an ethanol/benzene (1/2, *v*/*v*) solution in a Soxhlet extractor operating at 90 °C for 6 h. AL, CaCl_2_, ChCl, LA, sodium chlorite, acetic acid, 50% NaOH solution, tert-butanol, and sulfuric acid were purchased from Daejung Chemical & Metals Co., Ltd. (Siheung, Korea) and used without further purification. Commercial PCNFs were supplied by Cellulose Lab, Co., Ltd. (Fredericton, NB, Canada).

### 2.2. Deep Eutectic Solvent Pretreatment

The ChCl-based DES with LA was synthesized at a molar ratio of 1/1. The mixture of ChCl and LA was stirred at 80 °C until the mixture became a clear liquid. Then, the wood powder (2 g) was added to the DES (98 mL) and stirred at 400 rpm for 24 h at 120 °C and 130 °C. After DES treatment, the reactant was centrifuged at 4000× g for 20 min, and the supernatant and slurry were separated. The DES-insoluble residue was washed by vacuum filtration with a 1, 4-dioxane/water (4/1) solution.

### 2.3. Preparation of Cellulose Nanofibrils (CNFs)

The DES-treated products and commercial PCNFs were suspended at 1.0 wt% and pretreated using a high-speed blender at 30,000 rpm for 15 min. The samples were diluted at 0.1 wt% concentration and subjected to HPH (MN400BF, Micronox Co. Ltd., Sungnam, Korea). The pressure was set up to 20,000 psi, and the defibrillation procedure was repeated until the fifth pass.

### 2.4. Chemical Composition Analysis

#### 2.4.1. Klason Lignin Content

Lignin content in the DES-treated products was determined by the Klason method. The DES-treated samples (1 g) were added to a 72% sulfuric acid solution (15 mL) and stirred for 2 h at 20 ± 3 °C. Distilled water (560 mL) was subsequently added to the mixture to dilute sulfuric acid, and the residue was hydrolyzed in an autoclave at 120 °C for 1 h. The acid-insoluble residue was separated by vacuum filtration and washed with excess distilled water until the filtrate was pH-neutral. The lignin content was calculated by comparing the weight of the Klason lignin to the weight of the raw material.

#### 2.4.2. Determination of Cellulose and Hemicellulose Content

In order to determine the content of hemicellulose and cellulose, delignification was performed according to the Wise method [32]. Wood powder (10 g) was added to distilled water (600 mL) and then kept in a water bath at 80 °C for 20 min while stirring at 150 rpm. The delignification reaction was initiated by adding sodium chlorite (4 g) and acetic acid (800 µL) into the suspension, which was continuously stirred for 1 h. The same amount of sodium chlorite and acetic acid was added every hour, and the process was repeated seven times. The residue was then vacuum-filtrated and washed with distilled water several times until the pH was neutralized.

Then, the contents of hemicellulose and cellulose were measured from holocellulose by the following alkaline treatment. First, holocellulose (5 g) was added to a 17.5% NaOH solution (125 mL), and the reaction was performed for 30 min while stirring at 150 rpm at a temperature of 25 ± 3 °C. At the end of the reaction, 10% acetic acid (125 mL) was added to the solution for neutralization. Then, the reactant was vacuum-filtrated and washed with distilled water several times to obtain pure cellulose. The hemicellulose and cellulose contents were calculated by comparing the cellulose weight to the weight of the holocellulose.

### 2.5. Preparation of Wet-Spun Filament

**Figure 1:**
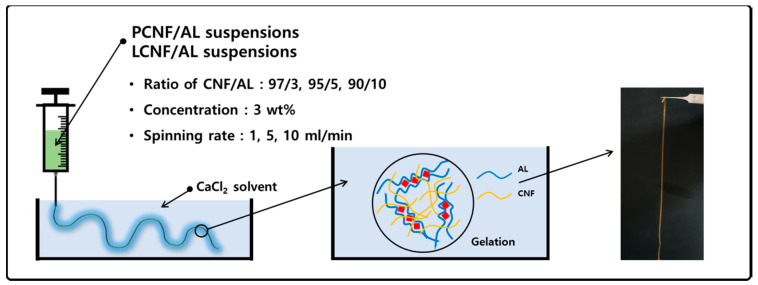
Scheme of composite filament preparation from pure cellulose nanofibril (PCNF)/alginate (AL) and lignocellulose nanofibril (LCNF)/AL suspension via wet spinning.

AL (3 g) was dissolved in distilled water (97 g) at 60 °C for 12 h under constant stirring at 200 rpm to prepare an AL solution of 3 wt% concentration. The prepared AL solution was mixed with PCNFs and LCNFs at a ratio of 97/3, 95/5, and 90/10 (CNF/AL). The concentration of the CNF/AL spinning suspension was adjusted to 3 wt%. The prepared spinning suspensions were placed into a syringe with a 24 G needle (outer diameter: 0.56 mm; inner diameter: 0.30 mm), and then wet-spun in 2 wt% CaCl_2_ solution at a spinning rate of 1, 5, and 10 mL/min (Figure 1). The wet-spun composite filaments were removed from the CaCl_2_ solution and immersed in acetone for solvent exchange. The filaments were air-dried at 25 ± 3 °C for 3 h.

### 2.6. Morphological Characterization

To observe the morphology of CNFs using a scanning electron microscope (SEM), the sample was prepared using the following method. The CNF suspensions were first diluted to 0.001 wt% and then sonicated using an ultrasonicator (VCX130PB, Sonics & Materials, Inc, Newtown, CT, USA) for 90 s. Next, the suspensions were vacuum-filtered through a polytetrafluoroethylene (PTFE) membrane filter. The PTFE filter was immersed and kept in tert-butanol for 30 min for solvent exchange. This solvent exchange was repeated five times to replace water with tert-butanol. The solvent-exchanged CNFs were freeze-dried using a freeze dryer (FDB-5502, Operon Co. Ltd., Gimpo, Korea) at −55 °C for 3 h to prevent the aggregation of the CNFs.

The morphological characteristics of the CNFs and wet-spun composite filaments were observed using an SEM (S-4800, Hitachi, Ltd., Tokyo, Japan) at the Central Laboratory of the Kangwon National University. The samples were placed on aluminum stubs and iridium coated to a thickness of 2 nm using a sputter coater (EMACE600, Leica Microsystems, Ltd., Wetzlar, Germany). The morphologies were observed at an accelerating voltage of 1 kV with a working distance of 8.5 mm.

### 2.7. Specific Surface Area

The CNF samples for the specific surface area measurement were prepared using the following method. The CNF suspension was aliquoted into a centrifuge tube and centrifuged at 40,000× g for 20 min, and then the supernatant was drained via decantation. Then, tert-butanol was poured into the centrifuge tube and mixed with the sediment using a vortex mixer. This solvent exchange process was repeated 15 times to remove the water and maintain the nanoscale morphology of the CNFs. The centrifuged samples were freeze-dried at −55 °C for 12 h. The specific surface area of the obtained nanocellulose powder was measured using a sorption analyzer (BELSORP-Max, BEL Japan Inc., Osaka, Japan) by N_2_ adsorption at 77 K.

### 2.8. Water Retention Value

A PTFE membrane filter (pore size: 0.2 µm) was placed on a glass filter (pore size: 10 µm) and embedded in a 50 mL centrifugation tube. The CNF suspensions were placed on the membrane filters and centrifuged at 2000× g for 15 min in a swing rotor. The LCNF pancake was carefully removed from the PTFE membrane filter. The samples were dried at 105 °C to a constant weight. The water retention value was calculated using Equation (1):

(1) $$WRV\;\left(\%\right)=\frac{m_{1}-m_{2}}{m_{2}}\times 100$$

where *WRV* is the water retention value, *m*_1_ is the LCNF weight after centrifugation, and *m*_2_ is the weight of the dried sample.

### 2.9. 2D X-ray Diffraction (2D XRD)

To analyze the orientation index of the wet-spun composite filaments, 2D XRD analysis was carried out using a 2D-XRD analyzer (Bruker D8 Discover with Vantec 500 detector, Bruker Corp., Billerica, MA, USA) with Cu-Kα radiation (λ = 1.5406 Å) at the Korea Basic Science Institute, Daegu Center. The 2D XRD patterns were recorded with an accelerating voltage of 40 kV, an accelerating current of 40 mA, and a beam size of 1.0 mm. A total of 40 composite filaments were bundled together to obtain sufficient intensity. From the 2D XRD patterns, the orientation index (α) of the filaments was calculated using the following Equation (2) by azimuthal breadth analysis:

(2) $$\mathrm{Orientation}\;\mathrm{index}\;\left(\alpha \right)=\left(180-\beta_{c}\right)/180$$

where *βc* is the half-width of the azimuthal direction of the equatorial reflection of the (200) plane obtained from the 2D XRD patterns.

### 2.10. Mechanical Properties

The wet-spun composite filaments were kept in a thermohygrostat at a relative humidity of 30% to minimize the influence of variation in relative humidity on the tensile strength. The mechanical properties were measured with a universal testing machine by applying a load cell of 5 N, gauge length of 10 mm, and cross-head speed of 3 mm/min. Five specimens of each sample were tested, and the average values of tensile strength, elastic modulus, and elongation at break are reported.

### 2.11. Contact Angle

The contact angle of filaments was measured using distilled water that was dropped onto the surface by a 1 mL syringe. The measurement was repeated 3 times, and the average contact angle was measured using an optical tensiometer (CAM 101, KSV Instruments Ltd., Espoo, Finland).

## 3. Results and Discussion

### 3.1. Chemical Composition of LCNFs

Table 1 shows the chemical composition of LCNFs prepared by ChCl-LA DES at 120 °C and 130 °C for 24 h. As the reaction temperature increased, the contents of Klason lignin and hemicellulose decreased. The increase in the temperature of DES can weaken the interaction between anions and cations in the DES and generate free anions and cations. Free anions and cations can accelerate the dissolution of lignocellulose components. Zhang et al. [33] investigated the effect of pretreatment temperature using ChCl-based DES with LA on the residue recovery of corn cob. It was reported that as the reaction temperature was increased in the range of 70–110 °C, the residue recovery after DES treatment decreased due to the dissolution of lignocellulose components. In particular, as the temperature increases, the solubility of lignin increases; this can be demonstrated by the increase in lignin extraction with increasing temperature. Sample codes were named LCNF-9 and LCNF-4 depending on the Klason lignin content.

### 3.2. Morphological Characteristics of PCNF, LCNF-4, and LCNF-9

**Figure 2:**
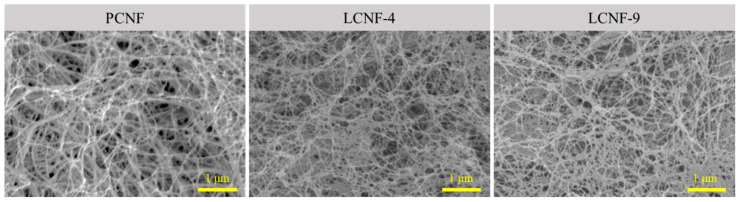
Morphological characteristics of PCNF, LCNF-4, and LCNF-9.

Figure 2 shows the morphological characteristics of PCNF, LCNF-4, and LCNF-9. In all samples, the CNFs have uniform morphologies indicating a diameter of 15–20 nm. The average diameter, specific surface area, and water retention value of the PCNF, LCNF-4, and LCNF-9 are summarized in Table 2. The average diameter of PCNF, LCNF-4, and LCNF-9 was about 17.4 nm, 15.3 nm, and 18.2 nm, respectively. LCNF-9 has a thicker diameter, indicating a lower value of specific surface area than PCNF and LCNF-4. This is because the lignin can decrease the efficiency of mechanical defibrillation [15]. Water retention value was in the order of PCNF > LCNF-4 > LCNF-9. The higher lignin content resulted in the decrease in water retention value due to the hydrophobic nature of lignin.

### 3.3. Wet-Spinnability

**Figure 3:**
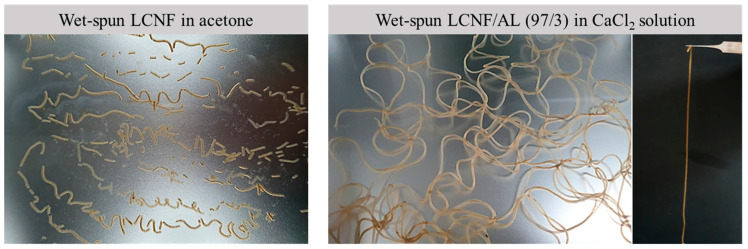
The effect of AL addition in LCNF suspension on wet-spinnability.

In order to improve the wet-spinnability of LCNFs into filaments, AL was used as a binder. The effect of AL addition on the wet-spinnability of LCNF suspensions in a coagulation bath is shown in Figure 3. The LCNF suspension was wet-spun in a coagulation bath with acetone for filament formation. However, the filament could not be formed and was easily broken. This was due to weak hydrogen bonding between the LCNFs because of the presence of hydrophobic lignin. In order to improve wet-spinnability, AL was added to the LCNF suspension at a ratio of 97/3 (LCNF/AL) as a binder. The spinning suspension of LCNF/AL was wet-spun in a coagulation bath with CaCl_2_ solution. The wet-spinnability was improved significantly as a result of the addition of AL, which formed a gel in the coagulation bath with CaCl_2_ solution due to cross-linkages with Ca^2+^; this contributed to the improvement of filament formation.

### 3.4. Morphological Characteristics of Wet-Spun Composite Filament

**Figure 4:**
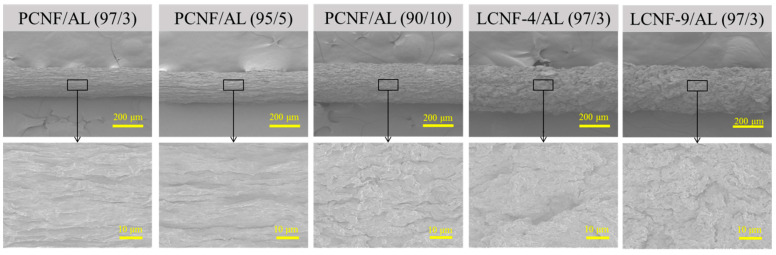
Morphological characteristics of wet-spun composite filament from PCNF/AL, LCNF-4/AL, and LCNF-9/AL with different ratios of CNF/AL at spinning rate of 10 mL/min.

Figure 4 shows the morphologies of the wet-spun composite filaments from PCNF/AL, LCNF-4/AL, and LCNF-9/AL with different ratios of CNF/AL at a spinning rate of 10 mL/min. In the PCNF/AL samples, aggregated CNF and AL formed filaments with uniform thickness. This shows the tendency of the diameter to increase with increasing AL. The wet-spun LCNF/AL composite filament had a larger diameter with a rougher surface than the PCNF/AL samples.

The dependence of the diameter of the wet-spun composite filament on the ratio of CNF/AL and spinning rate is summarized in Table 3. In all samples, the average diameter decreased as the spinning rate increased. The increase in the AL content in filaments caused an increase in the average diameter. At the same CNF/AL ratio and spinning rate, the average diameter of the composite filaments was in the order of PCNF/AL < LCNF-4/AL < LCNF-9/AL. It was found that the average diameter increased with increasing lignin content, and the filaments from LCNFs showed a rough surface. Park et al. [34] prepared filaments via wet-spinning from PCNF, HCNF, and LCNF suspensions. They also reported that the filaments made of LCNF had a larger diameter with a rougher surface than the filaments made of HCNFs or PCNFs.

### 3.5. X-ray Diffraction of Wet-Spun Composite Filaments

**Figure 5:**
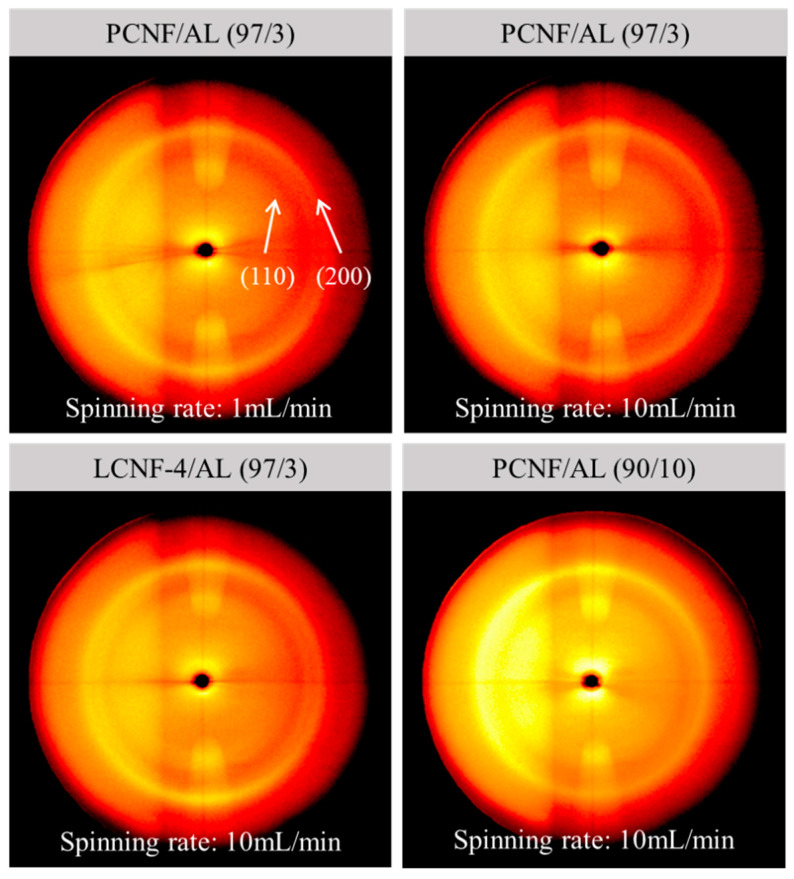
2D XRD patterns of wet-spun composite filaments made of PCNF/AL and LCNF-4/AL with different AL contents and spinning rates.

**Figure 6:**
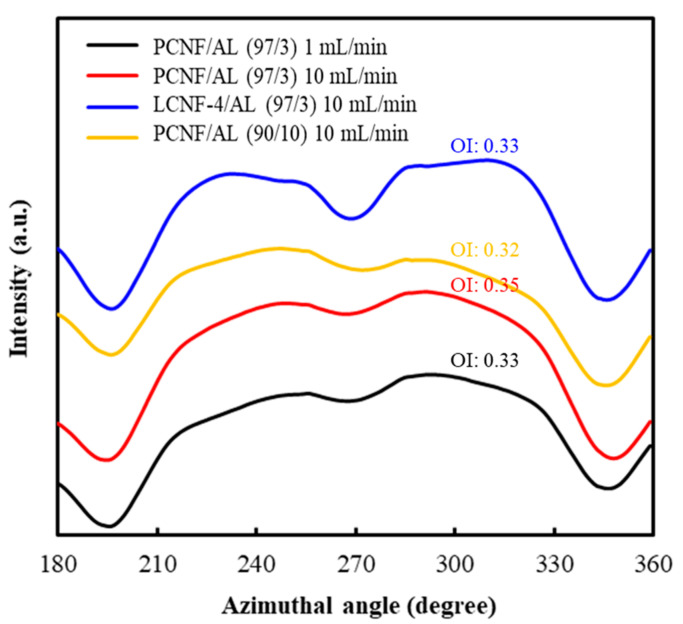
Azimuthal profiles of the (200) reflections from 2D XRD patterns of the wet-spun composite filaments made of PCNF/AL and LCNF-4/AL with different AL contents and spinning rates. Note: orientation index (OI).

Figure 5 shows 2D XRD patterns of wet-spun composite filaments from PCNF/AL and LCNF-4/AL with different AL contents and spinning rates. The wet-spun composite filaments showed a typical peak for cellulose I, corresponding to the (110) and (200) planes. Generally, it was known that filaments containing well-orientated nanocellulose show an arc pattern in 2D XRD patterns. However, the obtained wet-spun composite filaments made of PCNF/AL and LCNF-4/AL indicated a ring pattern. This ring pattern might be due to the low orientation of PCNFs and LCNFs in the presence of AL in the composite filaments. The orientation index of the composite filaments was calculated from the azimuthal profiles of the (200) reflections in the 2D XRD patterns, as shown in Figure 6 and summarized in Table 4. Some studies have reported that the orientation indexes of wet-spun nanocellulose filament were in the range of 0.6–0.8 [34,35], but the orientation indexes of the obtained wet-spun filament made of PCNF/AL and LCNF/AL were in the range of 0.33–0.35. This phenomenon might be due to the gelled AL, which has extremely high viscosity and interferes with the orientation of CNFs in the filament. In PCNF/AL filaments, as the spinning rate was increased from 1 mL/min to 10 mL/min, the orientation index tended to increase. This indicated that the increase of spinning rate can improve the shearing force on CNFs, resulting in the increased orientation index of CNFs.

### 3.6. Tensile Properties

**Figure 7:**
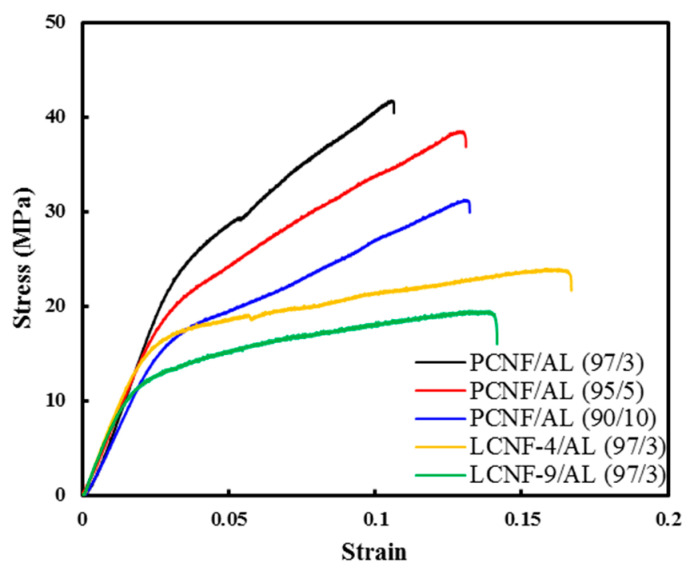
Stress–strain curves of wet-spun composite filament from PCNF/AL, LCNF-4/AL, and LCNF-9/AL with different AL content (spinning rate: 10 mL/min).

**Figure 8:**
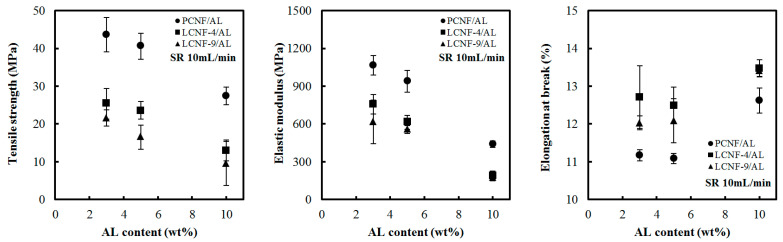
Tensile strength, elastic modulus, and elongation at break of wet-spun composite filament from PCNF/AL, LCNF-4/AL, and LCNF-9/AL with different AL content (spinning rate: 10 mL/min).

Figure 7 and Figure 8 show the tensile strength, elastic modulus, and elongation at break of the composite filaments from PCNF/AL, LCNF-4/AL, and LCNF-9/AL with different AL contents at a spinning rate of 10 mL/min. In all samples, as the content of AL increased, the tensile strength and elastic modulus decreased. The deterioration in tensile strength and elastic modulus resulting from AL addition is due to the lower strength and elastic modulus of AL compared to CNFs [36]. At the same AL content, the tensile strength and elastic modulus were in the order of PCNF/AL > LCNF-4/AL > LCNF-9/AL. The higher lignin content in the composite filaments resulted in deterioration of the tensile strength and elastic modulus. Hydrophobic lignin decreased the hydrogen bonding between the nanofibrils, resulting in a decrease in the tensile strength and elastic modulus. Tensile strength of composite filaments made of LCNF-9/AL and LCNF-4/AL was in the range of 10–30 MPa. In the previous study, wet-spun LCNF filaments had tensile strength in the range of 80–150 MPa, which is much stronger than LCNF/AL composite filaments. Since AL fiber has a low tensile strength of about 20 MPa [37], AL addition can deteriorate the strength of filaments. Elongation at break of the wet-spun composite filament was in the range of 1–4%, which increased with the increase in the content of AL.

**Figure 9:**
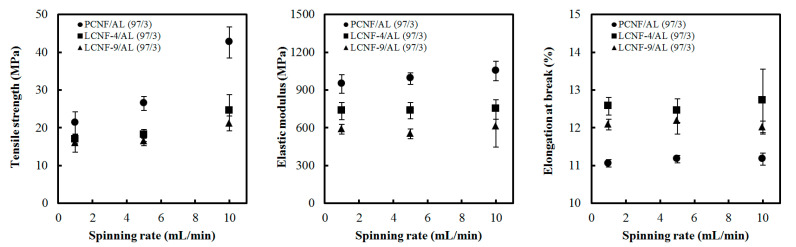
Tensile strength, elastic modulus, and elongation at break of wet-spun composite filaments from PCNF/AL, LCNF-4/AL, and LCNF-9/AL with a CNF/AL ratio of 97/3 at different spinning rates.

Figure 9 shows tensile strength, elastic modulus, and elongation at break of composite filaments made of PCNF/AL (97/3), LCNF-4/AL (97/3), and LCNF-9/AL (97/3) at different spinning rates. In all samples, the tensile strength and elastic modulus increased with increasing spinning rates. This phenomenon might be due to the increase in the orientation index of CNFs by the increase in the spinning rate (Table 4), indicating the improvement of tensile properties. Park et al. [34] investigated the effect of spinning rate and orientation index on mechanical properties of wet-spun filament made of LCNF, HCNF, and PCNF. They reported that the increase in the spinning rate can increase the orientation index, resulting in the improvement of tensile strength and elastic modulus. As the orientation increases, the cellulose crystals in CNFs are aligned along the fiber axis, enhancing the mechanical properties [38]. Elongation at break of the composite filaments was in the range of 1–3%.

**Figure 10:**
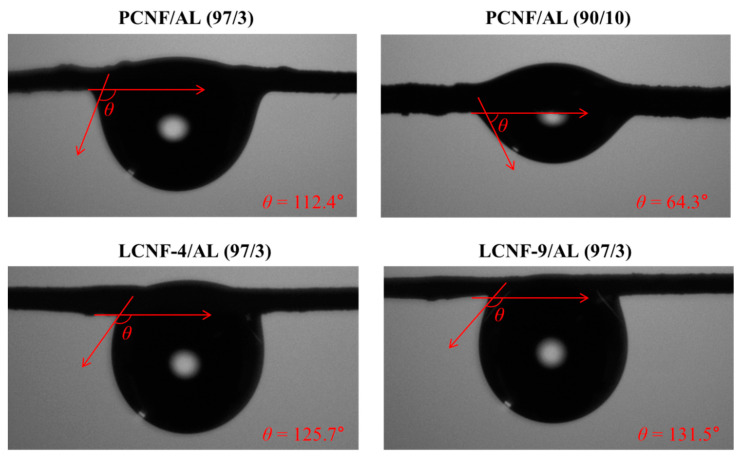
Contact angles of wet-spun composite filaments from PCNF/AL, LCNF-4/AL, and LCNF-9/AL.

Figure 10 shows contact angles of wet-spun composite filaments from PCNF/AL, LCNF-4/AL, and LCNF-9/AL. In the samples containing 3 wt% AL, the contact angle was in the order of LCNF-9 > LCNF-4 > PCNF. This phenomenon can be explained by the existence of hydrophobic lignin. Some parameters, such as roughness, affected the contact angle, but chemical composition would be the more important parameter, because of the same composite filament making process. The composite filament from LCNF-9/AL indicated a higher value of contact angle because it contains higher hydrophobic lignin [39]. In PCNF/AL composite filaments, as AL content increased, the contact angle was decreased. This may be because AL can be soluble in water and has very strong hydrophilicity.

## 4. Conclusions

LCNFs were prepared using ChCl-LA (1/1) DES pretreatment at 120°C and 130°C for 24 h with lignin contents of 9.3% and 3.6%, respectively. The average diameters of PCNFs, LCNF-4 (lignin 3.6%), and LCNF-9 (lignin 9.3%) were approximately 17.4 nm, 15.3 nm, and 18.2 nm, respectively. As the lignin content of the CNFs increased, the specific surface area and water retention values decreased. Composite filaments were prepared from PCNFs, LCNF-4, and LCNF-9 through wet-spinning, and AL was used as a binder to improve wet-spinnability. As the AL content increased in the composite filaments, the average diameter increased and the orientation index decreased. The increase in AL improved the wet-spinnability of CNFs but deteriorated the tensile properties. The increase in the spinning rate resulted in an increase in the orientation index, which improved the tensile strength and elastic modulus. These results demonstrate that AL contributes to improving the wet-spinnability of LCNFs. It is expected that the wet-spun LCNF/AL composite filaments can be utilized in biomedical materials including dressings, wound healing, and hemostasis.

## Figures and Tables

**Table 1:** Chemical composition of LCNFs prepared by ChCl-LA DES pretreatment.

Raw Material	Reaction Temperature (°C)	Chemical Composition		
		Cellulose	Hemicellulose	Klason Lignin
	-	44.8	23.8	31.4
LCNF-9	120	80.5	10.2	9.3
LCNF-4	130	91.7	4.7	3.6

**Table 2:** Average diameter, specific surface area, and water retention value of PCNF, LCNF-4, and LCNF-9.

Sample	Average Diameter (nm)	Specific Surface Area (m^2^/g)	Water Retention Value (%)
PCNF	17.4 ± 2.1	180.7	706.6
LCNF-4	15.3 ± 0.4	178.3	583.3
LCNF-9	18.2 ± 0.3	136.7	515.4

**Table 3:** Average diameter of wet-spun composite filaments from PCNF/AL, LCNF-4/AL, and LCNF-9/AL at different ratios of CNF/AL and spinning rates.

Sample	Ratio of CNF/AL	Spinning Rate (mL/min)	Average Diameter (μm)
PCNF/AL	97/3	1	289.4 ± 5.2
		5	273.3 ± 7.2
		10	247.0 ± 3.9
	95/5	10	263.8 ± 6.0
	90/10	10	334.7 ± 3.0
LCNF-4/AL	97/3	1	351.0 ± 9.8
		5	324.7 ± 10.3
		10	302.4 ± 8.5
	95/5	10	338.7 ± 10.3
	90/10	10	386.2 ± 9.5
LCNF-9/AL	97/3	1	388.1 ± 5.3
		5	368.2 ± 4.7
		10	342.8 ± 6.2
	95/5	10	373.7 ± 5.5
	90/10	10	410.0 ± 5.8

**Table 4:** Orientation index of wet-spun composite filaments from PCNF/AL, LCNF-4/AL, and LCNF-9/AL at different ratios of CNF/AL and spinning rates.

Sample	Ratio of CNF/AL	Spinning Rate (mL/min)	Orientation Index
PCNF/AL	97/3	1	0.33
		5	0.34
		10	0.35
	95/5	10	0.33
	90/10	10	0.32
LCNF-4/AL	97/3	1	0.33
LCNF-9/AL	97/3	1	0.33

## Data Availability

Data is available upon request from the corresponding author(s).

## References

[1] Isikgor F.H., Becer C.R. (2015). Lignocellulosic biomass: A sustainable platform for the production of bio-based chemicals and polymers. Polym. Chem..

[2] Ahorsu R., Medina F., Constantí M. (2018). Significance and Challenges of Biomass as a Suitable Feedstock for Bioenergy and Biochemical Production: A Review. Energies.

[3] Van Sebille E., Wilcox C., Lebreton L., Maximenko N., Hardesty B.D., Van Franeker J.A., Eriksen M., Siegel D., Galgani F., Law K.L. (2015). A global inventory of small floating plastic debris. Environ. Res. Lett..

[4] Cole M., Lindeque P., Halsband C., Galloway T.S. (2011). Microplastics as contaminants in the marine environment: A review. Mar. Pollut. Bull..

[5] Hurley R., Woodward J., Rothwell J.J. (2018). Microplastic contamination of river beds significantly reduced by catchment-wide flooding. Nat. Geosci..

[6] Cheng H., Wang L. (2013). Lignocelluloses Feedstock Biorefinery as Petrorefinery Substitutes. Biomass Now—Sustainable Growth and Use.

[7] Klemm D., Kramer F., Moritz S., Lindström T., Ankerfors M., Gray D., Dorris A. (2011). Nanocelluloses: A new family of nature-based materials. Angew. Chem. Int. Ed..

[8] Lee S.H., Kim H.J., Kim J.C. (2019). Nanocellulose applications for drug delivery: A review. J. For. Environ. Sci..

[9] Abe K., Iwamoto S., Yano H. (2007). Obtaining Cellulose Nanofibers with a Uniform Width of 15 nm from Wood. Biomacromolecules.

[10] de Barros R.D.R.O., de Sousa Paredes R., Endo T., da Silva Bon E.P., Lee S.H. (2013). Association of wet disk milling and ozonolysis as pretreatment for enzymatic saccharification of sugarcane bagasse and straw. Bioresour. Technol..

[11] Hu Z., Zhai R., Li J., Zhang Y., Lin J. (2017). Preparation and Characterization of Nanofibrillated Cellulose from Bamboo Fiber via Ultrasonication Assisted by Repulsive Effect. Int. J. Polym. Sci..

[12] Siró I., Plackett D. (2010). Microfibrillated cellulose and new nanocomposite materials: A review. Cellulose.

[13] Zhang L., Tsuzuki T., Wang X. (2015). Preparation of cellulose nanofiber from softwood pulp by ball milling. Cellulose.

[14] Lee S.H., Chang F., Inoue S., Endo T. (2010). Increase in enzyme accessibility by generation of nanospace in cell wall supramolecular structure. Bioresour. Technol..

[15] Park C.-W., Han S.-Y., Namgung H.-W., Seo P.-N., Lee S.-Y., Lee S.-H. (2017). Preparation and Characterization of Cellulose Nanofibrils with Varying Chemical Compositions. Bioresources.

[16] Lundahl M.J., Klar V., Wang L., Ago M., Rojas O.J. (2016). Spinning of Cellulose Nanofibrils into Filaments: A Review. Ind. Eng. Chem. Res..

[17] Araki J., Miyayama M. (2020). Wet spinning of cellulose nanowhiskers; fiber yarns obtained only from colloidal cellulose crystals. Polymer.

[18] Gao Q., Wang J., Liu J., Wang Y., Guo J., Zhong Z., Liu X. (2021). High mechanical performance based on the alignment of cellulose nanocrystal/chitosan composite filaments through continuous coaxial wet spinning. Cellulose.

[19] Kim H.C., Kim D., Lee J.Y., Zhai L., Kim J. (2019). Effect of Wet Spinning and Stretching to Enhance Mechanical Properties of Cellulose Nanofiber Filament. Int. J. Precis. Eng. Manuf. Technol..

[20] Walther A., Timonen J.V., Díez I., Laukkanen A., Ikkala O. (2011). Multifunctional High-Performance Biofibers Based on Wet-Extrusion of Renewable Native Cellulose Nanofibrils. Adv. Mater..

[21] Abdollahi M., Alboofetileh M., Rezaei M., Behrooz R. (2013). Comparing physico-mechanical and thermal properties of alginate nanocomposite films reinforced with organic and/or inorganic nanofillers. Food Hydrocoll..

[22] Norajit K., Kim K.M., Ryu G.H. (2010). Comparative studies on the characterization and antioxidant properties of biodegradable alginate films containing ginseng extract. J. Food Eng..

[23] Lagopati N., Pavlatou E.A. (2020). Advanced Applications of Biomaterials Based on Alginic Acid. Am. J. Biomed. Sci. Res..

[24] Qin Y., Jiang J., Zhao L., Zhang J., Wang F. (2018). Applications of alginate as a functional food ingredient. Biopolymers for Food Design.

[25] Raus R.A., Nawawi W.M.F.W., Nasaruddin R.R. (2021). Alginate and alginate composites for biomedical applications. Asian J. Pharm. Sci..

[26] Szekalska M., Puciłowska A., Szymańska E., Ciosek P., Winnicka K. (2016). Alginate: Current Use and Future Perspectives in Pharmaceutical and Biomedical Applications. Int. J. Polym. Sci..

[27] Lee K.Y., Mooney D.J. (2012). Alginate: Properties and biomedical applications. Prog. Polym. Sci..

[28] Liu J., Liu Y., Miao D., Sui S., Zhang C., Zhu P. (2018). Gelation Modification of Alginate Nonwoven Fabrics. Fibers Polym..

[29] Rousseau I., Le Cerf D., Picton L., Argillier J.F., Muller G. (2004). Entrapment and release of sodium polystyrene sulfonate (SPS) from calcium alginate gel beads. Eur. Polym. J..

[30] Liu J., Zhang R., Ci M., Sui S., Zhu P. (2019). Sodium alginate/cellulose nanocrystal fibers with enhanced mechanical strength prepared by wet spinning. J. Eng. Fibers Fabr..

[31] Ureña-Benavides E.E., Kitchens C.L. (2012). Cellulose nanocrystal reinforced alginate fibers—Biomimicry meets polymer processing. Mol. Cryst. Liq. Cryst..

[32] Wise L.E. (1946). Chlorite holocellulose, its fractionation and bearing on summative wood analysis and on studies on the hemicelluloses. Pap. Trade.

[33] Zhang C.-W., Xia S.-Q., Ma P.-S. (2016). Facile pretreatment of lignocellulosic biomass using deep eutectic solvents. Bioresour. Technol..

[34] Park C.W., Park J.S., Han S.Y., Lee E.A., Kwon G.J., Seo Y.H., Gwon J.G., Lee S.Y., Lee S.H. (2020). Preparation and characteristics of wet-spun filament made of cellulose nanofibrils with different chemical compositions. Polymers.

[35] Iwamoto S., Isogai A., Iwata T. (2011). Structure and Mechanical Properties of Wet-Spun Fibers Made from Natural Cellulose Nanofibers. Biomacromolecules.

[36] Benselfelt T., Engström J., Wågberg L. (2018). Supramolecular double networks of cellulose nanofibrils and algal polysaccharides with excellent wet mechanical properties. Green Chem..

[37] Park J.-S., Park C.-W., Han S.-Y., Lee E.-A., Cindradewi A.W., Kim J.-K., Kwon G.-J., Seo Y.-H., Youe W.-J., Gwon J. (2021). Preparation and Properties of Wet-Spun Microcomposite Filaments from Various CNFs and Alginate. Polymers.

[38] Kafy A., Kim H.C., Zhai L., Kim J.W., Kang T.J. (2017). Cellulose long fibers fabricated from cellulose nanofibers and its strong and tough characteristics. Sci. Rep..

[39] Seo P.N., Han S.Y., Park C.W., Lee S.Y., Kim N.H., Lee S.H. (2019). Effect of alkaline peroxide treatment on the chemical compositions and characteristics of lignocellulosic nanofibrils. Bioresources.

